# Automatic Classification of Normal and Cancer Lung CT Images Using Multiscale AM-FM Features

**DOI:** 10.1155/2015/230830

**Published:** 2015-09-15

**Authors:** Eman Magdy, Nourhan Zayed, Mahmoud Fakhr

**Affiliations:** Computer and Systems Department, Electronic Research Institute, Giza 12611, Egypt

## Abstract

Computer-aided diagnostic (CAD) systems provide fast and reliable diagnosis for medical images. In this paper, CAD system is proposed to analyze and automatically segment the lungs and classify each lung into normal or cancer. Using 70 different patients' lung CT dataset, Wiener filtering on the original CT images is applied firstly as a preprocessing step. Secondly, we combine histogram analysis with thresholding and morphological operations to segment the lung regions and extract each lung separately. Amplitude-Modulation Frequency-Modulation (AM-FM) method thirdly, has been used to extract features for ROIs. Then, the significant AM-FM features have been selected using Partial Least Squares Regression (PLSR) for classification step. Finally, *K*-nearest neighbour (*K*NN), support vector machine (SVM), naïve Bayes, and linear classifiers have been used with the selected AM-FM features. The performance of each classifier in terms of accuracy, sensitivity, and specificity is evaluated. The results indicate that our proposed CAD system succeeded to differentiate between normal and cancer lungs and achieved 95% accuracy in case of the linear classifier.

## 1. Introduction

Computed Tomography (CT) has outperformed conventional radiography in the screening of lungs because it generates very detailed high-resolution images and can show early-stage lesions that are too small to be detected by conventional X-ray. CT has been widely used to detect numerous lung diseases, including pneumoconiosis, pneumonia, pulmonary edema, and lung cancer [1]. Early detection of diseases is very crucial for treatment planning. However, it is considered one of the most challenging tasks performed by radiologists due to the huge amount of data generated by CT scan. Therefore, computer-aided diagnostic (CAD) systems are needed to assist radiologists in the analysis and evaluation of CT scans.

A CAD system analyzes medical images in several steps: first a preprocessing step for noise reduction and enhancing the image quality and then segmentation step to differentiate region of interest (ROI) from other structures in the image. After segmentation, different features such as geometrical, textural, and statistical features are extracted. Finally, a classification/evaluation step is done to evaluate and diagnose the ROI based on extracted features.

Many efforts have been made to provide computer-aided diagnosis for lung images. Lung segmentation is a necessary step; it has progressed from manual tracing to semiautomated to fully automated segmentation. Here, some automated lung segmentation studies are presented [2–9]. Other studies present content-based image retrieval (CBIR) systems for lung images [10–15]. Earlier work in classification of lung cancer includes the work of Patil and Kuchanur [16] and Kuruvilla and Gunavathi [17] that used artificial neural networks to classify lung cancer images based on the features extracted from lung segmented images. Nevertheless, Patil and Kuchanur used geometrical features for classification and achieved only 83% accuracy of classification. And Kuruvilla and Gunavathi used statistical parameters as features for classification and achieved accuracy of 93.3%. Another work by Depeursinge et al. [18] classified different lung tissue patterns using discrete wavelet frames combined with gray-level histogram features. However, the main limitation of this work was the lack of resolution in scales with the decomposition, along with required feature weighting while merging features from different origins.

In this paper, we propose a CAD system for analysis, automatic segmentation, and classification of lung images into normal or cancer from CT dataset. The system is based on the multiscale Amplitude-Modulation Frequency-Modulation (AM-FM) approach. The lungs are firstly segmented from CT images and next left and right lungs are separated individually to be analyzed over a filterbank. Then, the AM-FM features are extracted and reduced for the classification step. Different classifiers are used to classify the images and the performance of each classifier has been evaluated.

## 2. Materials and Methods

**Figure 1:**
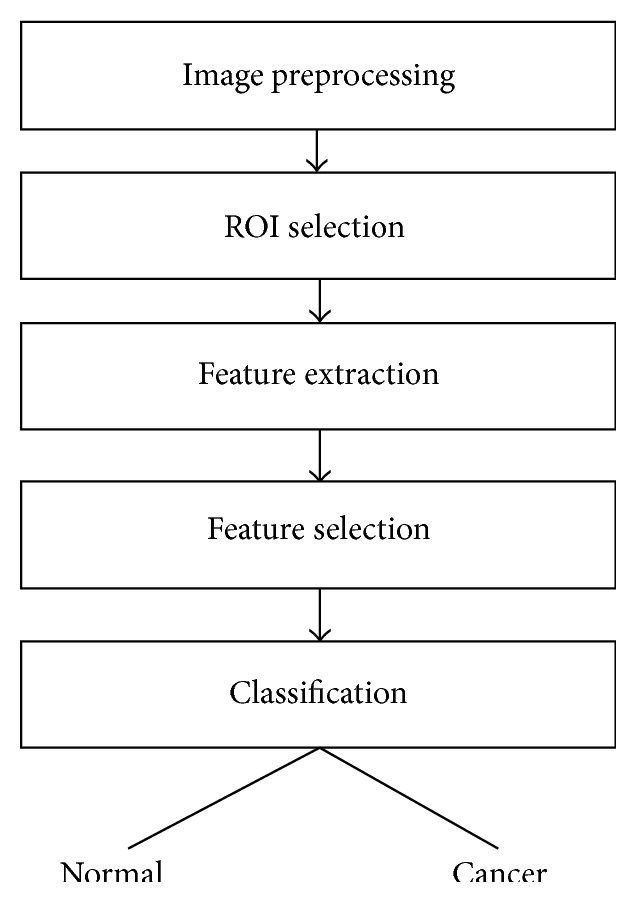
Block diagram of the proposed fully automated CAD system.

Figure 1 shows the main block diagram of our proposed fully automated CAD system. As seen in this figure, the system is composed of five main steps: image preprocessing, select region of interest (ROI), feature extraction using AM-FM approach, feature selection to find the significant features, and finally a classification. The details of each step are discussed in the following sections.

### 2.1. Dataset

**Figure 2:**
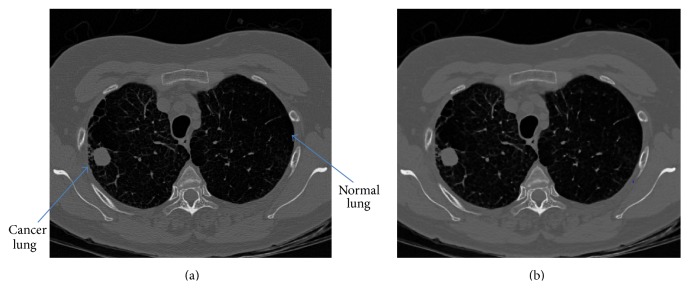
Image preprocessing: (a) original image and (b) filtered image.

Data used in this research were obtained from The Cancer Imaging Archive (TCIA) sponsored by the SPIE, NCI/NIH, AAPM, and the University of Chicago [19]. A dataset of 83 CT images from 70 different patients was included. All images have a size of 512 × 512 pixels and are stored in Digital Imaging and Communication in Medicine (DICOM) format. An example of dataset is shown in Figure 2(a). In this figure, the right lung is abnormal as it has a cancer (the rounded gray shape), while the left lung is a normal one. For each lung CT image, we separate the left lung from the right lung automatically (as discussed later in ROI Selection), and each separated lung is labelled as normal or cancer based on the dataset information.

### 2.2. Image Preprocessing

The objective of preprocessing step is to remove unwanted noise and enhance image quality. We have used a Wiener filter to remove noise while preserving the edges and fine details of lungs. The filter size of 3 × 3 is selected to avoid oversmoothing of the image. The result of Wiener filtering is shown in Figure 2(b).

Wiener filtering [20] is based on estimating the local mean and variance from a local neighborhood of each pixel. Then, it creates pixel-wise linear filtering using these estimates:

(1) $$\begin{array}{c} F\left(\;m,n\;\right)=\mu +\frac{\sigma^{2}-v^{2}}{\sigma^{2}}\left(\;I\left(\;m,n\;\right)-\mu \;\right), \end{array}$$

where *I* and *F* denote the original and filtered images, respectively, *μ* and *σ* denote the mean and variance of a local neighborhood, respectively, and *v* is the noise variance.

### 2.3. ROI Selection

Lung segmentation is a necessary step for any lung CAD system. We perform automatic segmentation of the lungs using successive steps. Then, the resulting segmented image is used to extract each lung separately (ROIs), producing two images: one for the left lung and the other for the right lung.

In the CT image, air appears in a mean intensity of approximately −3024 Hounsfield units (HU), and the lung tissue is in the range of −910 HU to −500 HU, while other structures are above −500 HU. The goal of segmentation step is to separate the lungs from both background and nonlung regions. To accomplish this, we propose a hybrid technique resulting from a combination of histogram analysis, thresholding, and morphological operations for automatic lung segmentation.

**Figure 3:**
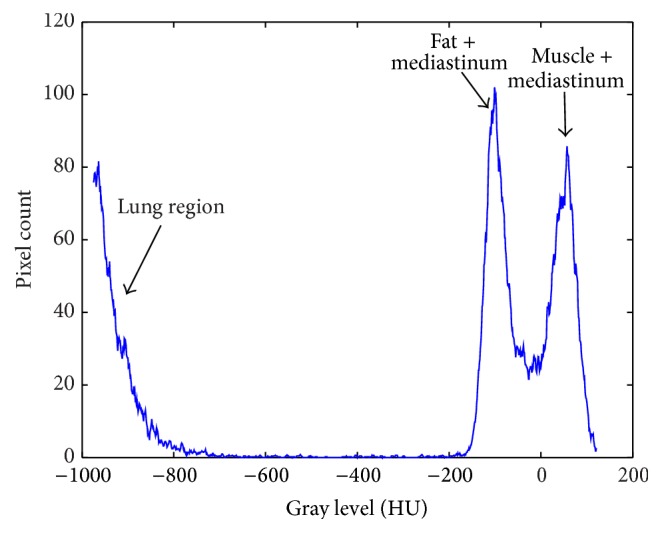
Histogram of the Wiener-filtered image.

To simplify the segmentation process, the thorax region is firstly segmented from the background. A gray-level distribution (histogram) of the Wiener-filtered image is used to identify different regions in the image. The histogram has one peak corresponding to lung region and another two peaks for fat and muscle of thorax region and lung mediastinum. In addition, there is a spike at −3024 HU corresponding to background pixels.  Figure 3 shows all peaks except for the background spike.

The threshold value is then computed from this histogram according to the following equation:

(2) $$\begin{array}{c} T=\frac{I_{\text{FM}}-I_{L}}{2}+I_{L}, \end{array}$$

where *I*_*L*_ denotes the peak intensity value of lung region and *I*_FM_ denotes the average intensity value of fat/muscle peaks.

**Figure 4:**
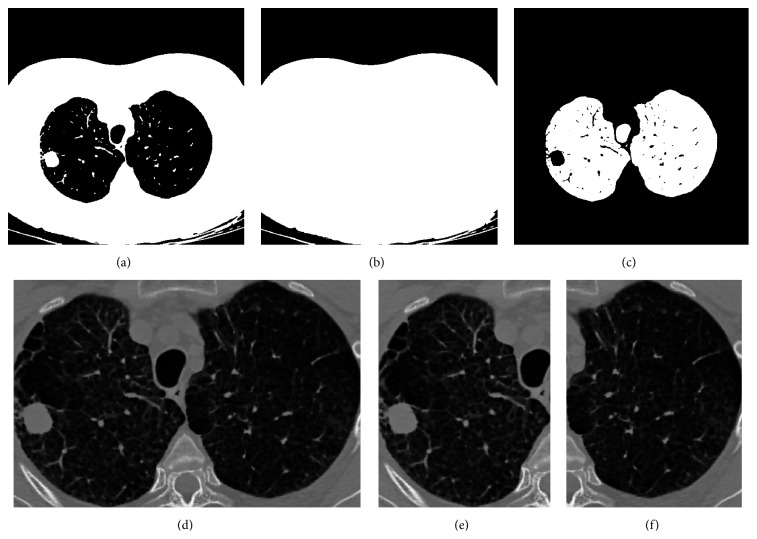
Segmentation and ROI selection. (a) Thorax binary image. (b) Filled image. (c) Lung segmentation. (d) Rectangular ROI of both lungs. (e) Right lung (cancer). (f) Left lung (normal).

Then, a binary image (Figure 4(a)) for segmented thorax region is created where the pixels with gray level greater than the selected threshold are set to “one” and other pixels are “zero.”

After the thorax region is segmented, we perform a filling operation to fill the holes inside the binary image, so that the pixel values of lungs change from zero to one and produce a filled image (Figure 4(b)). Finally, the thorax binary image is subtracted from the filled image to obtain the segmented lungs as shown in Figure 4(c).

Once the image of lung segmentation is obtained, we used it to locate the left and right lungs in the filtered image, as seen in Figure 4(d). Then, this image is divided into two images for both lungs separately, each one covering the region of the lung as shown in Figures 4(e) and 4(f). From the 83 CT images, we obtained 166 different lung images after each image division, where 83 of them are normal lung images and the other 83 images are cancer lung images.

### 2.4. Feature Extraction

In this step, we apply the Amplitude-Modulation Frequency-Modulation (AM-FM) modeling techniques to extract features from lung images that will be used further in classification. A lot of research work has been made on AM-FM models [21–23].

#### 2.4.1. AM-FM Methods

AM-FM is a technique that models nonstationary signals. Unlike Fourier transforms that provide the frequency content of signal, AM-FM methods provide pixel-based information in terms of instantaneous amplitude (IA), instantaneous frequency (IF), and instantaneous phase (IP). And it does not have the main limitation found on wavelet when used to segment the lungs which was the lack of resolution in scales with the decomposition.

In 2D AM-FM model, a nonstationary image is represented by a sum of AM-FM components as [22]

(3) $$\begin{array}{c} I\left(\;k_{1},k_{2}\;\right)=\overset{M}{\underset{n=1}{\sum }}a_{n}\left(\;k_{1},k_{2}\;\right)\cos\varphi_{n}\left(\;k_{1},k_{2}\;\right), \end{array}$$

where *n* = 1,2,…, *M* denotes the different AM-FM harmonics, *a*_*n*_(*k*_1_, *k*_2_) denotes the instantaneous amplitude functions (IA), and *φ*_*n*_(*k*_1_, *k*_2_) denotes the instantaneous phase functions (IP).

For each AM-FM component, the instantaneous frequency (IF) is defined as the gradient of phase  ∇*φ*_*n*_(*k*_1_, *k*_2_) = (∂*φ*_*n*_(*k*_1_, *k*_2_)/∂*k*_1_, ∂*φ*_*n*_(*k*_1_, *k*_2_)/∂*k*_2_). Here, the AM-FM demodulation problem is to estimate the IA, IF, and IP for the given input image.

In this work, AM-FM demodulation is achieved in several steps. First, we extend the input image to an analytic image by adding an imaginary part equal to the 2D Hilbert transform of the image [21]. Given a real-valued image *I*(*k*_1_, *k*_2_), the analytic image *I*_AS_(*k*_1_, *k*_2_) is calculated as follows:

(4) $$\begin{array}{c} I_{\text{AS}}\left(\;k_{1},k_{2}\;\right)=I\left(\;k_{1},k_{2}\;\right)+jH_{2\text{D}}\left[\;I\left(\;k_{1},k_{2}\;\right)\;\right], \end{array}$$

where *H*_2D_ denotes a two-dimensional extension of one-dimensional Hilbert transform.

Then, the analytic image is processed through a collection of band-pass filters (filterbank) (to be discussed in the next subsection) in order to isolate the AM-FM components.

And, from each filter response, we can estimate the IA and IP straightforwardly using these following equations:

(5) a^k1,k2=IASk1,k2,φ^k1,k2=tan−1⁡imagIASk1,k2realIASk1,k2.

To estimate the IF, we used a variable spacing local linear phase (VS-LLP) method as described in [23].

#### 2.4.2. Filterbank Design

**Figure 5:**
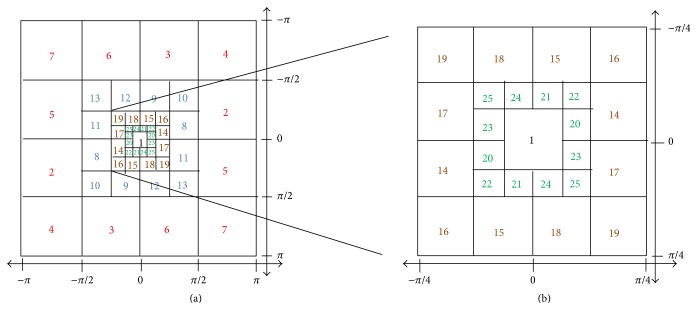
Four-scale filterbank [22]. (a) Complete frequency range of the filterbank. (b) Zoom on the low frequency filters (to be easily readable).

The purpose of multiscale filterbank is to isolate the AM-FM image components in model (3) prior to performing demodulation. Here, we use a four-scale filterbank developed by Murray [22] (see Figure 5).

In Figure 5, the frequency range of filterbank is depicted. Filter 1 is a low-pass filter (LPF), filters 2–7 are high frequency filters (H), filters 8–13 are medium frequency filters (M), filters 14–19 are low frequency filters (L), and filters 20–25 are very low frequency filters (VL). It can be noticed that the bandwidth is decreased by a factor of 1/2 for each added scale.

In this paper, we used different combinations of scales to extract the dominant AM-FM features. Here are the combinations used: (1) VL, L, M, and H; (2) LPF; (3) VL; (4) L; (5) M; (6) LPF, VL, L, M, and H; (7) LPF, VL; (8) VL, L; (9) L, M; (10) M, H; and (11) H. And, for each combination of scales, we estimate the IA, IP, and |IF| using (5) and the equations in [23].

#### 2.4.3. Histogram Processing

For each combination of scales, we produce a histogram for AM-FM estimates: IA, IP, and |IF|. And all the computed histograms are normalized so that the area of each histogram is equal to one. Then, for each combination of scales, we create a 96-bin feature vector from the IA, IP, and |IF| histograms, with 32 bins for IA, 32 bins for IF magnitude, and 32 bins for IP (centered at the maximum value). Therefore, each image produces 11 feature vectors corresponding to the 11 combinations of scales. We need to obtain a combined feature vector for each case by selecting the optimal and signification features from all over scales. Thus, we use Partial Least Squares Regression (PLSR) to achieve that.

#### 2.4.4. Feature Selection

Feature selection is an important step that provides the significant features, which are used to differentiate between different classes accurately. We used Partial Least Squares Regression (PLSR) [24], which is a linear regression method that finds the relation between the predicted variables and observations. The regression problem is defined as

(6) $$\begin{array}{c} y=X\beta +\varepsilon , \end{array}$$

where *X* is an *n* × *p* matrix of the extracted AM-FM features (*n* is the number of images and *p* is the number of features) and *y* is an *n* × 1 vector of response or labels. We used label 0 for normal case and label 1 for abnormal case. *β* is *p* × 1 vector of the regression parameters and *ε* is *n* × 1 vector of the residuals.

**Figure 6:**
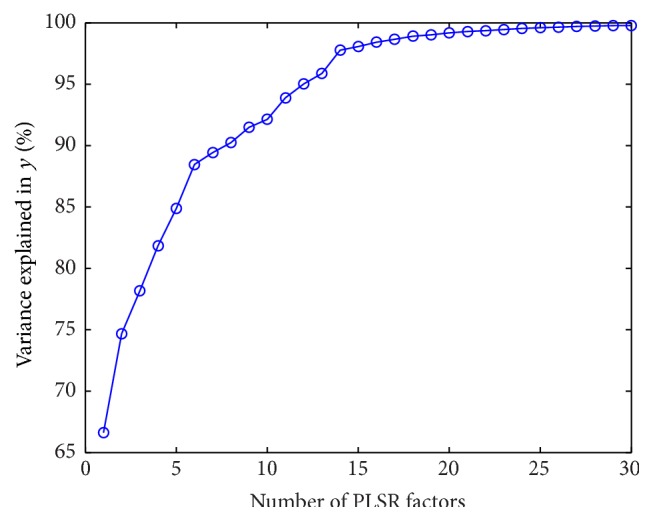
Plotting the number of PLSR factors with the cumulative variance percentage in the response variable *y*.

We apply PLSR to determine the optimal number of features to be used. We select the PLSR factors number that produces percentage of variance in response variable more than 90%. In Figure 6, we plot the percentage of variance in the response versus the number of PLSR factors. The plot shows that nearly 90% of the variance in *y* is given by the first eleven factors.

Once the optimal number of features is obtained, we form a feature vector to represent the selected features.

### 2.5. Classification

The final step of the proposed system is to correctly discriminate between normal and cancer lung images. The input to classification stage is the feature matrix from the previous step and the labeled vector (where 0 = normal and 1 = cancer).

Here, we have used four different classifiers: *K*-nearest neighbor (*K*NN) [25], support vector machine (SVM) [26], naïve Bayes [25], and linear classifier [25]. The basic idea of all of these classifiers depends on supervised learning; that is, each classifier takes a set of labeled images as a training set to build a model that is used further to assign new images (testing set) into classes. Out of 166 lung images, 100 images are selected as a training dataset and 66 other images are selected as a testing dataset.

## 3. Results and Discussion

The four classifiers are trained and their performance is evaluated with leave-*M*-out cross validation. We change the value of *M* to generate different sizes of testing and training sets, and, for each *M* value, the classification performance is evaluated by computing these different measures:

(7) Sensitivity%=TPTP+FN×100,Specificity%=TNFP+TN×100,Accuracy%=TP+TNTP+FN+TN+FP×100,

where TP, TN, FN, and FP denote true positive, true negative, false negative, and false positive, respectively [27].

**Figure 7:**
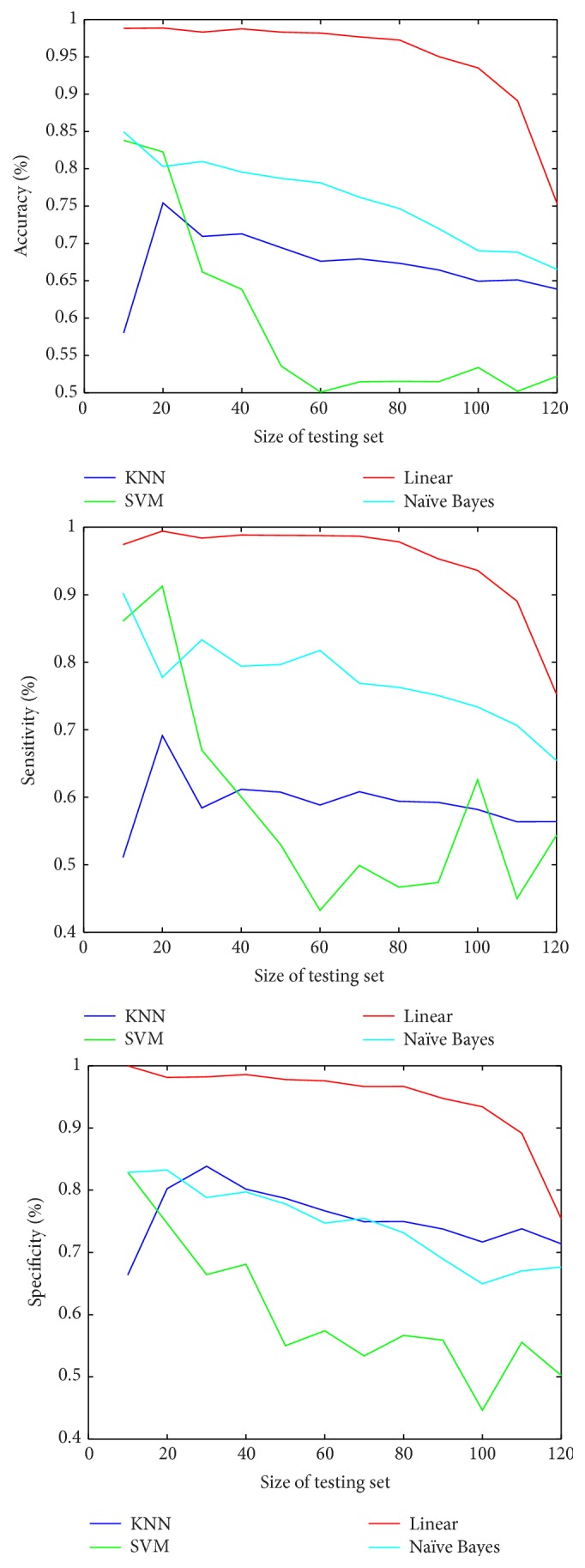
Comparison between accuracy, sensitivity, and specificity for the four classifiers with changing size of testing set.

Figure 7 shows the computed accuracy, sensitivity, and specificity, respectively, for the four classifiers with the change in size of testing set. It can be noticed that the classifiers performances in terms of accuracy, sensitivity, and specificity are much better in case of small size of the testing set (when the classifiers got trained with large size of the training set). However, the performances of all classifiers decrease with increasing testing set size. From this figure, it is easily observed that the performance of SVM classifier is the least stable with increasing testing set size, while the other three classifiers are more stable. Moreover, it can be concluded that the linear classifier is the best one to discriminate between normal and cancer lungs.

Table 1 summarizes the performance measures for the four classifiers when the size of training set relative to the testing set is 60% to 40% of the total dataset size, respectively (i.e., training set = 100 images and test set = 66 images). As shown in Table 1, the linear classifier gives the best classification with 95% accuracy, 94% sensitivity, and 97% specificity. On the other hand, the *K*NN classifier is the worst classifier achieving only 64% accuracy, 55% sensitivity, and 72% specificity.

It is worth noting that the proposed CAD system is developed using MATLAB R2010a on Intel Core i5, 2.5 GHz, CPU, 6 GB RAM, Windows 7 64-bit PC.

## 4. Conclusions

In this paper, we develop a lung CAD system that analyzes and automatically segments the lungs and classifies each lung to normal or cancer. The system consists of five main steps: preprocessing, ROI selection, feature extraction, feature selection, and classification. AM-FM approach has been used to extract new features in terms of IA, IP, and IF. And PLSR is then used to reduce the large number of features and select the optimal and significant ones. Four classifiers are used and the performance of each classifier has been evaluated. It has been found that the linear classifier was the best one to discriminate between normal and cancer lungs with 95% accuracy.

## Figures and Tables

**Table 1:** ****Classification performance measures for the four classifiers.

Classifier	Accuracy	Sensitivity	Specificity
KNN	64%	55%	72%
SVM	90%	85%	97%
Naïve Bayes	82%	82%	82%
Linear	95%	94%	97%
